# Supplementary material for: IgM N-glycosylation correlates with COVID-19 severity and rate of complement deposition
Source: Nat Commun. 2024 Jan 9;15:404. doi: 10.1038/s41467-023-44211-0 (PMC10776791; doi:10.1038/s41467-023-44211-0)
Supplement: Supplementary file 1 — Supplementary Information [file 41467_2023_44211_MOESM1_ESM.pdf]

Supplementary material for:

IgM N-glycosylation correlates with COVID-19 severity and rate of complement deposition

Supplemental Figure 1-6

Supplemental Table 1-10

Supplemental Figure 1

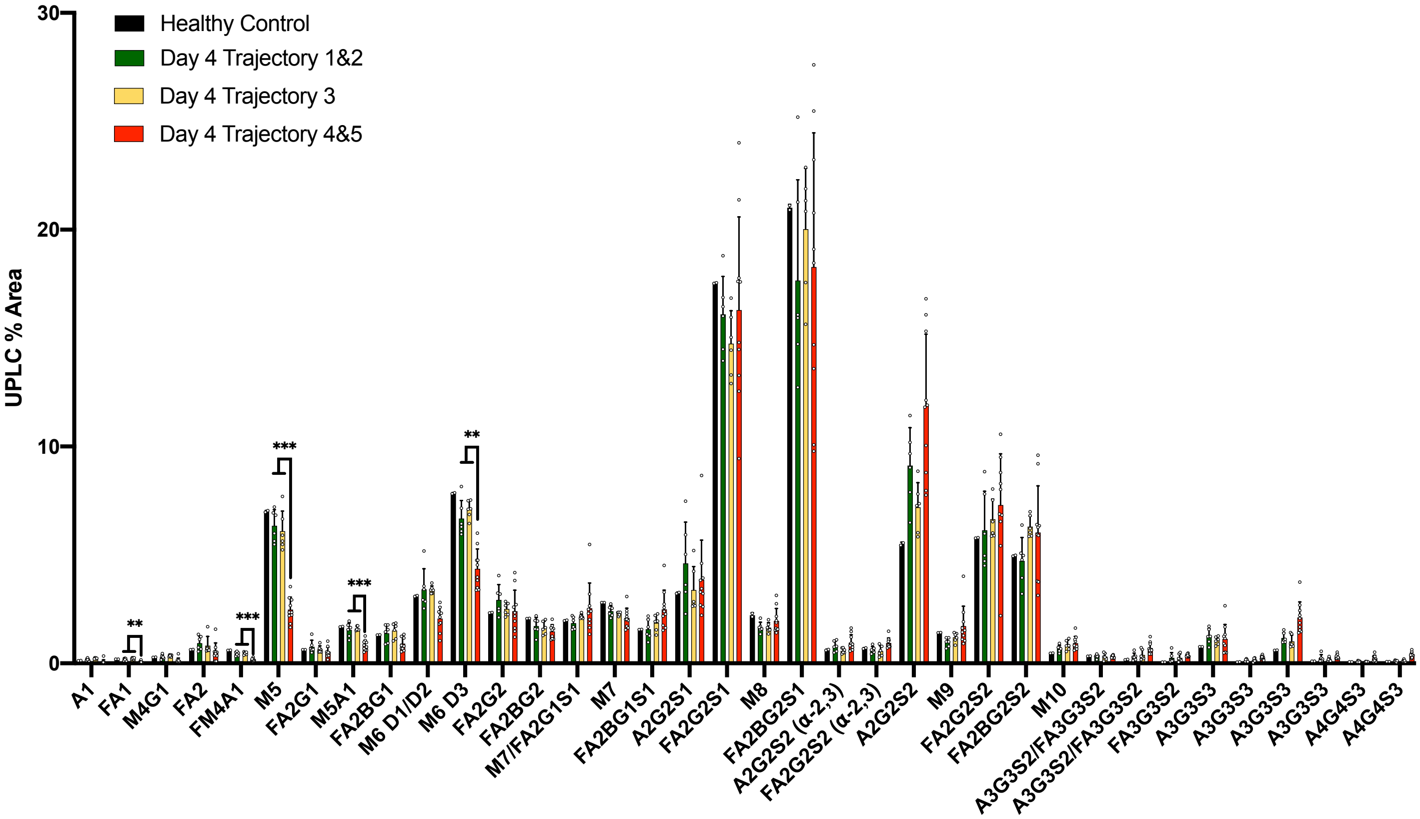

Supplemental Figure 1. IgM N-glycans labeled with Waters RapiFluor (RFMS) were profiled with UPLC-FLR-ESI-MS. Healthy Control (n=2), Day 4: Trajectory 1&2 (n=6), Trajectory 3 (n=6), and Trajectory 4&5 (n=10) COVID-19 hospitalized cohorts are presented by N-glycan. Statistical significance was determined using unpaired t-tests, graphed with mean values + S.D. **p <0.01, ***p<0.001.

Supplemental Figure 2

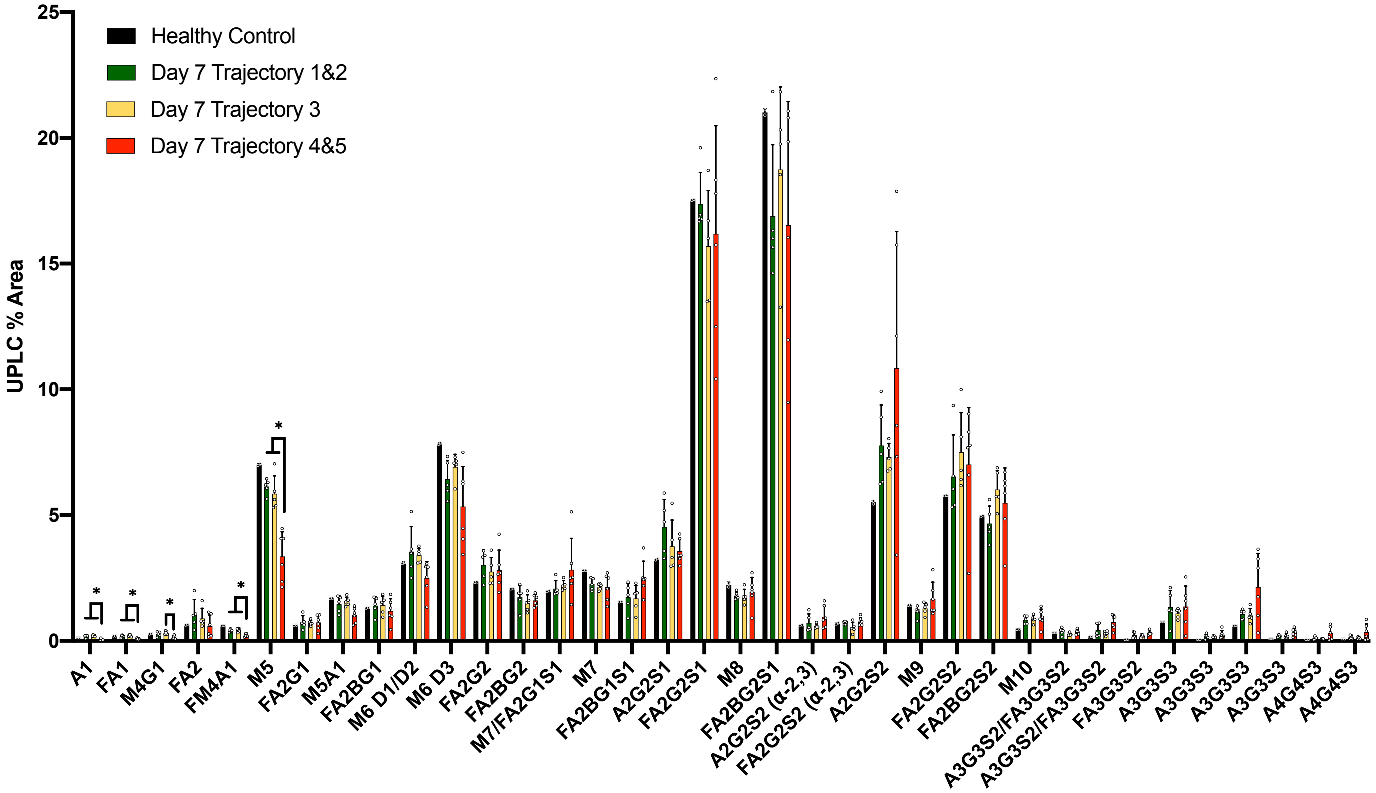

Supplemental Figure 2. IgM N-glycans labeled with Waters RapiFluor (RFMS) were profiled with UPLC-FLR-ESI-MS. Healthy Control (n=2), Day 7: Trajectory 1&2 (n=5), Trajectory 3 (n=5), and Trajectory 4&5 (n=6) COVID-19 hospitalized cohorts are presented by N-glycan mean values + S.D. Statistical significance was determined using unpaired t-tests *p <0.05.

Supplemental Figure 3

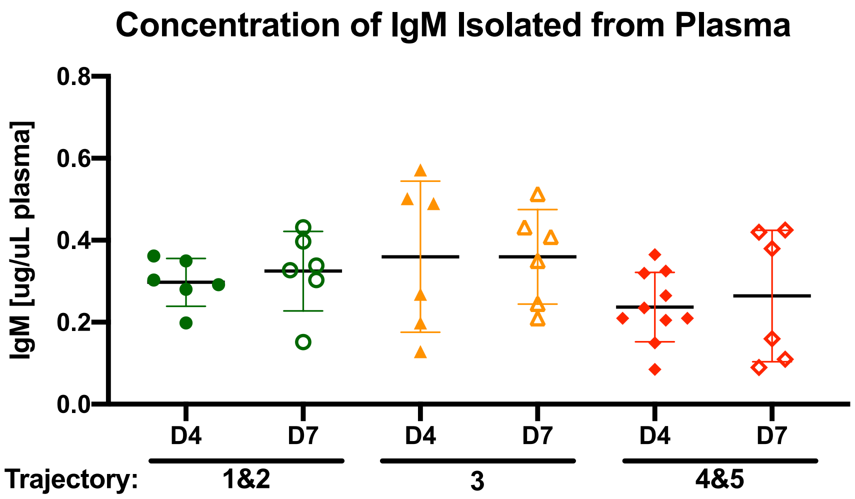

Supplemental Figure 3. The concentration of total IgM isolated from hospitalized COVID-19-infected patient cohort plasma across trajectories 1+2 (n=6), trajectory 3 (n=6), and trajectories 4+5 (n=10) on day 4 (D4) and across trajectories 1+2 (n=6), trajectory 3 (n=6), and trajectories 4+5 (n=6) day 7 (D7) are reported in units of μg IgM per μL plasma. A two-sided Mann-Whitney U test determined no statistically significant differences between trajectory cohorts or days of infection.

Supplemental Figure 4

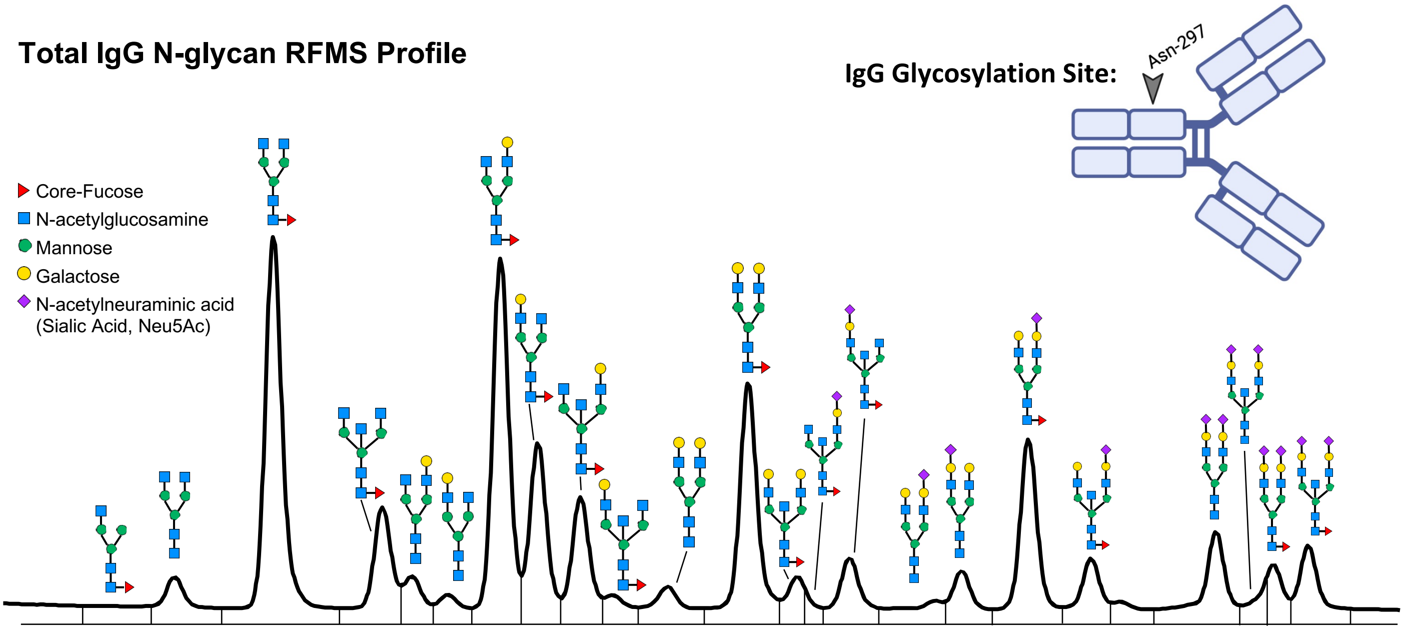

GP	1	2	3	4	5	6	7	8	9	10	11	12	13	14	15	16	17	18	19	20	21	22	23	24
**G0**	+	+	+	+																				
**G1**					+	+	+	+	+	+														
**G2**											+	+	+											
**S1**														+	+	+	+	+	+					
**S2**																					+	+	+	+
**Bisecting**				+					+	+			+	+	+				+			+		+
**Fucose**	+			+			+	+	+	+		+	+	+	+			+	+				+	+

Supplemental Figure 4 IgG N-glycans labeled with RapiFluor MS detected with UPLC-FLR-ESI-MS. IgG N-glycans are grouped by class: G0 refers to core diantennary N-glycans lacking galactose, G1 refers to core diantennary N-glycans with a single galactose, G2 refers to core diantennary N-glycans with two galactoses, S1 refers to diantennary N-glycans with a single sialic acid, S2 refers to diantennary N-glycans with two sialic acids, Bisecting refers to any N-glycan with a bisecting GlcNAc moiety, Fucose refers N-glycans with a core-fucose. Induvial glycan moieties are labeled on the upper left of the figure, with the conversed glycosylation site Asn-297 on IgG glycosylation site depicted in the upper right of the figure, created using BioRender.

Supplemental Figure 5

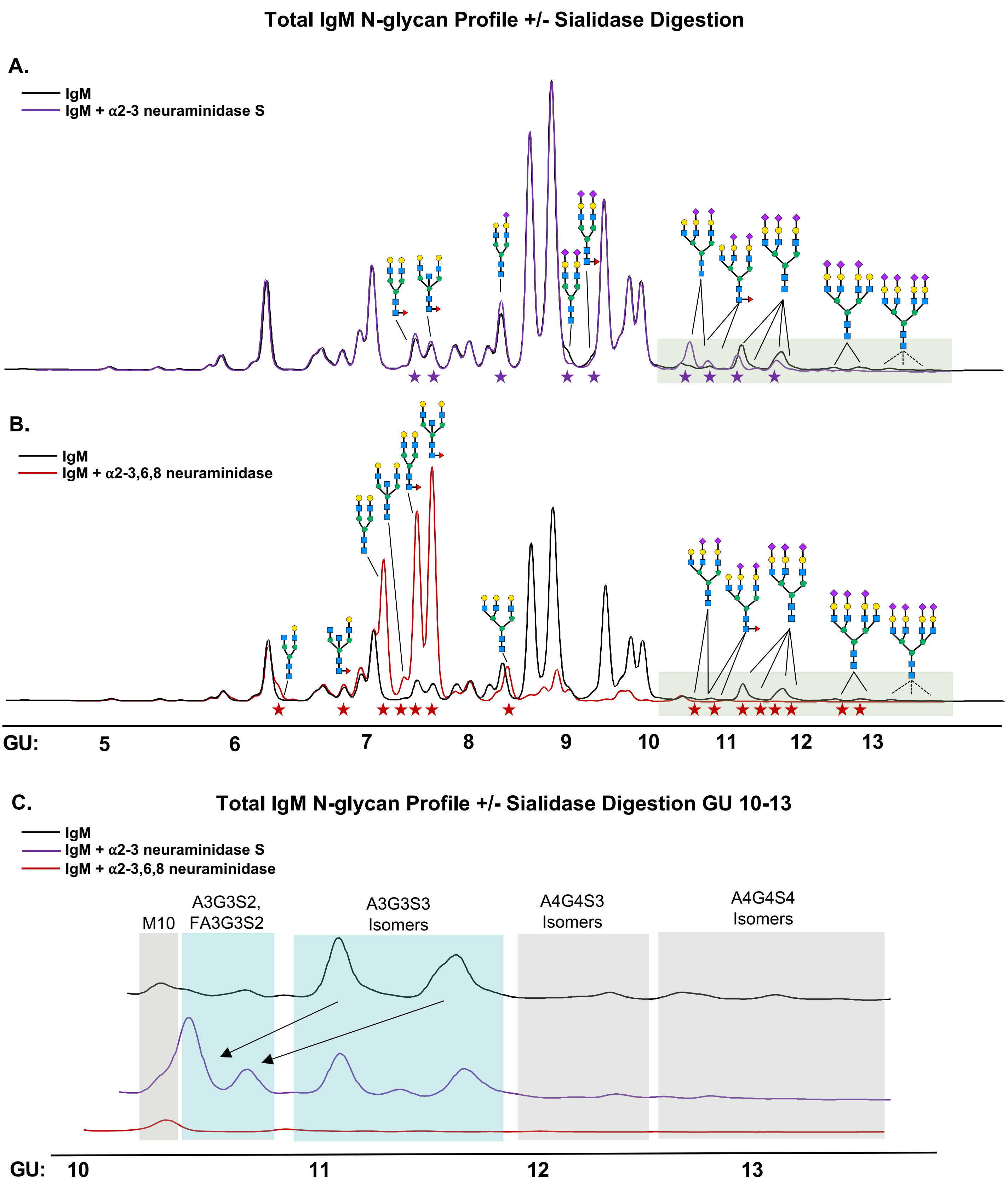

Supplemental Figure 5: IgM N-glycans digested with α2-3 linkage-specific neuraminidase reveal alpha-2,3 linked sialic acid on IgM. A) Neuraminidase S digested IgM N-glycan profile (purple) is overlaid with the un-digested IgM N-glycan profile (black). Purple stars indicate peak abundance shifts due to neuraminidase digestion. **B)** A non-linkage-specific neuraminidase digested IgM N-glycan profile (red) is overlaid with the un-digested IgM N-glycan profile (black). Red stars indicate peak abundance shifts due to non-specific neuraminidase digestion. **C)** Glucose Unit standard (GU) 10-13 IgM neuraminidase digestions are stacked to demonstrate the shifts observed following linkage-specific sialidase digestions on the A3G3S3 and A4G4S4 species.

Supplemental Figure 6

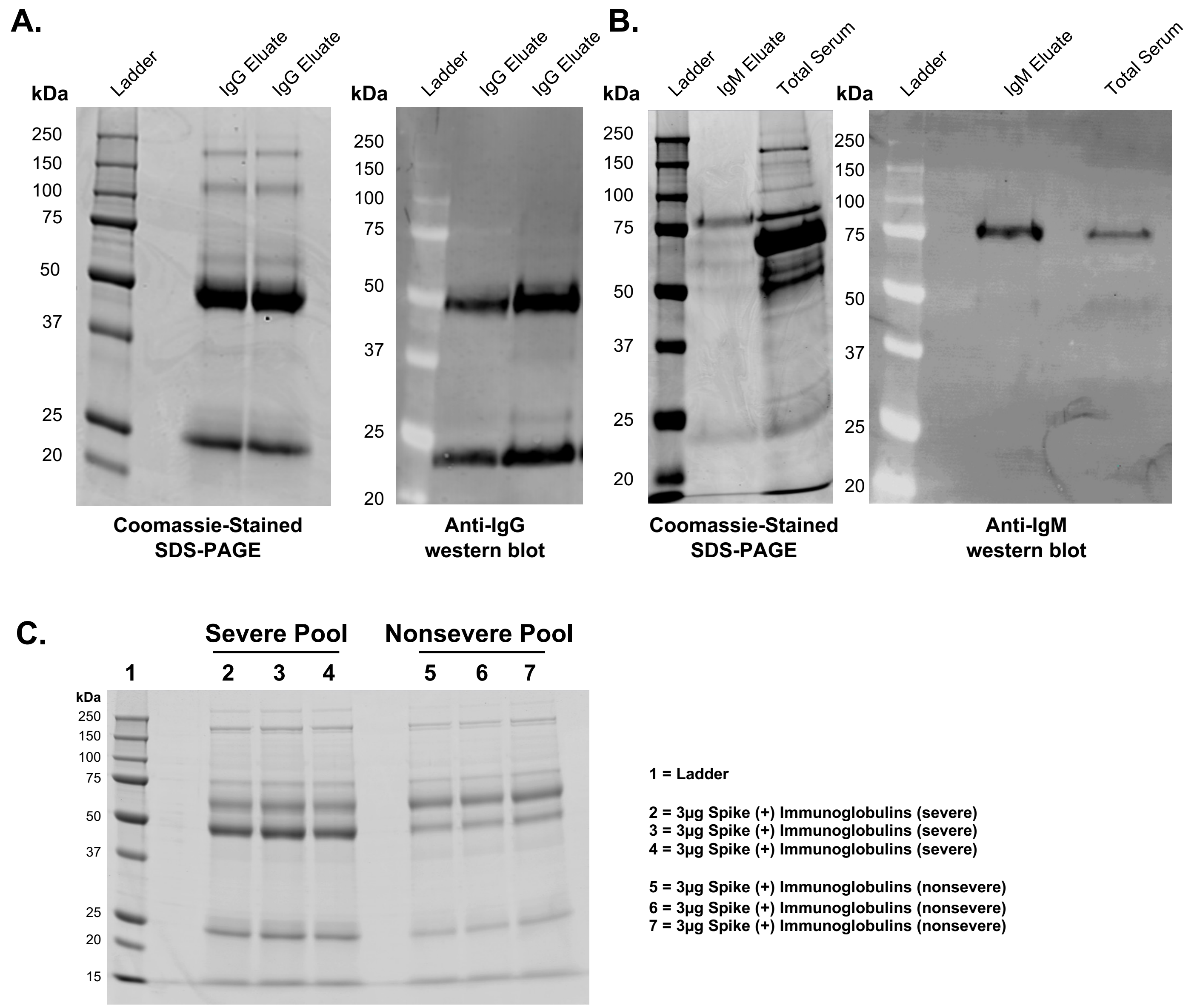

D.

		Severe				Nonsevere			
		75kDa		50kDa		75kDa		50kDa	
Protein	Description	S.C	% Glycoprotein	S.C	% Glycoprotein	S.C	% Glycoprotein	S.C	% Glycoprotein
sp|P01857|IGHG1_HUMAN	Immunoglobulin heavy constant gamma 1 {ECO:0000303|PubMed:11340299, ... Total	141	13.0%	2030	81.3%	87	13.7%	1211	71.4%
sp|P01871|IGHM_HUMAN	Immunoglobulin heavy constant mu {ECO:0000303|PubMed:11340299, ... Total	910	84.0%	52	2.1%	525	82.7%	45	2.7%
sp|P02768|ALBU_HUMAN	Albumin; Flags: Precursor; Total	413	-	212	-	350	-	306	-
sp|P01859|IGHG2_HUMAN	Immunoglobulin heavy constant gamma 2 {ECO:0000303|PubMed:11340299, ... Total	33	3.0%	415	16.6%	23	3.6%	441	26.0%
	Total Spectral Count (S.C.) of Glycosylated Protein	1084	100.0%	2497	100.0%	635	100.0%	1697	100.0%

Supplemental Figure 6. Assessment of immunoglobulin enrichment methods. A. Protein G IgG eluate analyzed using Coomassie-stained SDS-PAGE gel and anti-IgG western blot **B.** anti-IgM Agarose bead IgM eluate and total serum analyzed using Coomassie-stained SDS-PAGE gel and anti-IgM western blot **C.** Coomassie-stained SDS-PAGE gel of pooled severe and nonsevere Spike S1-specific immunoglobulin eluate **D.** The top 4 most abundant peptides identified by LC-MS/MS spectral count analysis of the 75kDa and 50kDa bands isolated from pooled severe and nonsevere patient spike S1-specific immunoglobulin eluate. S.C. = spectral counts

Supplemental Table 1

Supplemental Table 1. IgM N-glycans identified using mass spectrometry and retention time are listed in the table below. n.d. = not detected due to mass scan range of 500 to 1250 m/z.

Peak #	Observed Mass	Expected Mass	RFMS Glycan ID
1	712.9	713	A1
2	785.8	786	FA1
3	794	794	M4G1
4	887.8	887.9	FA2
5	867.3	867.4	FM4A1
6	773.88	773.8	M5
7	968.8	968.9	FA2G1
8	875.41	875.4	M5A1
9	1070.66	1070.4	FA2BG1
10	854.91	853.9	M6 D1/D2
11	854.89	853.9	M6 D3
12	1049/1013	1049.9	FA2G2
13	768.22/1151.43	768/1151.5	FA2BG2
14	935/1114.5	935.9/1114.4	M7/FA2G1S1
15	935	935.9	M7
16	811.24	811	FA2BG1S1
17	1122/1123	1122.4	A2G2S1
18	797.5/1195.6	797/1195.5	FA2G2S1
19	1016.89	1016.9	M8
20	865.2	865	FA2BG2S1
21	845.6	845.7	A2G2S2 *(α-2,3)*
22	894.7	894.3	FA2G2S2 *(α-2,3)*
23	845	845.7	A2G2S2
24	1098	1097	M9
25	894.6	894.3	FA2G2S2
26	962.29	962	FA2BG2S2
27	1179	1177.9	M10
28	967.5/1016	966.4/1015	A3G3S2/FA3G3S2
29	967.5/1016	966.4/1015	A3G3S2/FA3G3S2
30	1016	1015	FA3G3S2
31	1064	1063.4	A3G3S3 (6,6,6)
32	1064	1063.4	A3G3S3 (6,6,3)
33	1064	1063.4	A3G3S3 (6,3,3)
34	1064	1063.4	A3G3S3 (3,3,3)
35	1186	1185.1	A4G4S3 (6,6,6,6)
36	1186	1185.1	A4G4S3 (6,6,6,3)
37	n.d.	1282.1	Likely A4G4S4 (GU>13)
38	n.d.	1282.1	Likely A4G4S4 (GU>13)
39	n.d.	1282.1	Likely A4G4S4 (GU>13)

Supplemental Table 2

Supplemental Table 2. Linear regression results sorted by statistical significance total di-sialylated (S2) content detected from total plasma IgM N-glycan profiles were correlated to the transcriptomic expression of glycosyltransferases in PBMCs collected on day 0 of hospitalization, clinical data, the relative abundance of anti-nucleocapsid (anti-N) antibodies at visit 1 (day 0 hospitalization) and visit 2 (day 4 of hospitalization), viral RT-qPCR titers, and Luminex data from plasma samples. The coefficient of determination R2 was obtained from linear regression. $p < 0.05$ was considered statistically significant for all tests. Data was sorted by P-value, with $p<0.05$ highlighted in pink.

IgM S2 correlated to:	P value	R^2
anti-N IgA Visit 1	0.001	0.431
D-dimer	0.008	0.350
Creatinine	0.011	0.295
anti-N IgG Visit 1	0.016	0.267
anti-N IgM Visit 1	0.020	0.267
Glucose	0.030	0.224
BUN	0.037	0.208
ST3GAL4	0.051	0.177
IL-16	0.054	0.182
Ferritin	0.073	0.176
TMTC2	0.076	0.149
ST6GALNAC1	0.087	0.140
IL-18	0.099	0.137
MAN1A2	0.110	0.123
SCF	0.153	0.110
Potassium	0.186	0.090
ST6GALNAC4	0.193	0.083
Hemoglobin	0.201	0.085
TMTC1	0.297	0.054
ddCT_N2_RP Visit 2	0.298	0.063
MIF	0.301	0.056
PIGV	0.333	0.047
ST8SIA4	0.358	0.042
ST3GAL2	0.360	0.042
Height (cm)	0.366	0.041
MAN2A1	0.374	0.040
AST	0.379	0.041
POMT2	0.380	0.039
ST3GAL3	0.387	0.038
Serum bicarbonate (HCO3)	0.392	0.039
ST3GAL5	0.392	0.037
IFNy	0.404	0.037
ST6GALNAC2	0.432	0.031
MANEAL	0.452	0.029
MCP-1	0.464	0.029
MAN2A2	0.470	0.026
Albumin	0.472	0.029
MIP-1b	0.491	0.025
ENA-78	0.497	0.028
TNF-RII	0.498	0.025
BLC	0.500	0.026
ST8SIA5	0.504	0.023
IL-13	0.509	0.025
Sodium	0.519	0.022
ST3GAL1	0.528	0.020
TNF-a	0.532	0.022
ddCT_N1_RP Visit 2	0.535	0.023
Sex	0.539	0.019
EDEM2	0.548	0.018
IL-17A	0.571	0.019
ST8SIA1	0.572	0.016
HGF	0.586	0.017
DPY19L3	0.608	0.013
ALG9	0.617	0.013
MAN2C1	0.629	0.012
ALG12	0.636	0.011

IgM S2 correlated to:	P value	R^2
ST6GALNAC5	0.641	0.011
PIGB	0.645	0.011
EDEM3	0.649	0.011
ST6GAL2	0.650	0.011
DPM1	0.660	0.010
ST8SIA2	0.663	0.010
IL-6	0.674	0.010
ST6GALNAC6	0.684	0.008
MIP-3a	0.697	0.008
ST8SIA6	0.698	0.008
DPY19L4	0.709	0.007
TMTC4	0.715	0.007
EDEM1	0.728	0.006
ST6GAL1	0.728	0.006
CD30	0.735	0.006
IL-12p70	0.735	0.006
ST8SIA3	0.744	0.005
Age	0.749	0.005
CD40L	0.752	0.006
ALG1L	0.754	0.005
ALG2	0.758	0.005
ALG1	0.761	0.005
MCP-3	0.767	0.005
G-CSF	0.772	0.005
CRP	0.775	0.005
IL-20	0.781	0.004
Total bilirubin	0.785	0.004
IL-10	0.787	0.004
TSLP	0.788	0.004
ALG3	0.795	0.003
ST3GAL6	0.801	0.003
ST6GALNAC3	0.813	0.003
Weight (kg)	0.815	0.003
MAN2B2	0.816	0.003
MAN1A1	0.817	0.003
BMI (Calc)	0.823	0.003
TMTC3	0.831	0.002
MANEA	0.835	0.002
MAN1B1	0.858	0.002
MAN2B1	0.861	0.002
C20orf173	0.893	0.001
ITAC	0.899	0.001
PIGZ	0.903	0.001
ALG8	0.907	0.001
SDF2L1	0.928	0.000
ALT	0.935	0.000
DPY19L2	0.940	0.000
MAN1C1	0.944	0.000
ALG11	0.961	0.000
IL-15	0.961	0.000
DPY19L1	0.973	0.000
ALG1L2	0.976	0.000
MANBA	0.979	0.000
VEGF-A	0.991	0.000
POMT1	0.992	0.000
PIGM	0.996	0.000

Supplemental Table 3

Supplemental Table 3. Linear regression results sorted by statistical significance total mannose (Mann) content detected from total plasma IgM N-glycan profiles were correlated to the transcriptomic expression of glycosyltransferases in PBMCs collected on day 0 of hospitalization, clinical data, the relative abundance of anti-nucleocapsid (anti-N) antibodies at visit 1 (day 0 hospitalization) and visit 2 (day 4 of hospitalization), viral RT-qPCR titers, and Luminex data from plasma samples. The coefficient of determination R2 was obtained from linear regression. $p < 0.05$ was considered statistically significant for all tests. Data was sorted by P-value, with $p<0.05$ highlighted in pink.

IgM Mann correlated to:	P value	R^2
BUN	0.007	0.320
Potassium	0.008	0.317
ST3GAL4	0.008	0.302
Creatinine	0.010	0.299
MAN1A2	0.012	0.278
anti-N IgA Visit 1	0.020	0.254
TMTC2	0.032	0.209
D-dimer	0.041	0.224
IL-18	0.057	0.178
anti-N IgG Visit 1	0.059	0.175
IL-16	0.060	0.174
Glucose	0.061	0.172
ST8SIA4	0.082	0.144
ST6GALNAC2	0.105	0.126
Ferritin	0.110	0.143
ST6GALNAC4	0.127	0.113
SCF	0.135	0.120
MAN2A1	0.143	0.104
POMT2	0.143	0.104
MCP-1	0.147	0.107
ALG9	0.154	0.099
ST6GALNAC5	0.211	0.077
ENA-78	0.214	0.089
MCP-3	0.215	0.089
ST8SIA1	0.222	0.074
MIF	0.232	0.074
HGF	0.242	0.075
IL-6	0.261	0.070
IFNy	0.262	0.066
CD40L	0.262	0.073
Hemoglobin	0.280	0.061
PIGV	0.290	0.056
MIP-1b	0.303	0.056
ST8SIA5	0.307	0.052
ST8SIA2	0.317	0.050
MANEAL	0.321	0.049
IL-13	0.325	0.054
ST6GAL2	0.325	0.048
ST8SIA3	0.329	0.048
anti-N IgM Visit 1	0.333	0.052
ST3GAL2	0.334	0.047
IL-17A	0.335	0.055
Height (cm)	0.341	0.045
DPY19L2	0.349	0.044
ST3GAL5	0.353	0.043
Sodium	0.359	0.044
Sex	0.377	0.039
ST6GALNAC1	0.382	0.038
TNF-a	0.386	0.042
TMTC4	0.386	0.038
TMTC3	0.410	0.034
EDEM2	0.414	0.034
MANBA	0.444	0.030
CRP	0.449	0.034
MAN1C1	0.467	0.027
MANEA	0.476	0.026

IgM Mann correlated to:	P value	R^2
Serum bicarbonate (HCO3)	0.482	0.026
ALT	0.504	0.025
MAN1A1	0.518	0.021
IL-10	0.519	0.024
AST	0.532	0.021
ALG1L2	0.540	0.019
DPM1	0.541	0.019
DPY19L4	0.547	0.018
ST3GAL1	0.558	0.017
ALG11	0.560	0.017
C20orf173	0.573	0.016
TNF-RII	0.574	0.017
ST3GAL3	0.599	0.014
VEGF-A	0.600	0.015
Total bilirubin	0.601	0.016
POMT1	0.608	0.013
DPY19L1	0.609	0.013
ST6GALNAC3	0.615	0.013
SDF2L1	0.636	0.011
ALG12	0.650	0.011
BMI (Calc)	0.661	0.010
MAN2B1	0.670	0.009
ddCT_N2_RP Visit 2	0.674	0.011
MAN2B2	0.694	0.008
TMTC1	0.702	0.007
Age	0.707	0.007
ST8SIA6	0.713	0.007
BLC	0.729	0.007
ALG8	0.751	0.005
MAN2A2	0.781	0.004
ALG2	0.781	0.004
EDEM3	0.787	0.004
MAN2C1	0.804	0.003
IL-12p70	0.812	0.003
ddCT_N1_RP Visit 2	0.821	0.003
ST3GAL6	0.829	0.002
ALG1L	0.830	0.002
MAN1B1	0.837	0.002
TSLP	0.850	0.002
ALG3	0.863	0.002
PIGB	0.866	0.001
PIGZ	0.876	0.001
PIGM	0.897	0.001
EDEM1	0.899	0.001
ITAC	0.915	0.001
IL-15	0.917	0.001
MIP-3a	0.920	0.001
ALG1	0.922	0.000
DPY19L3	0.929	0.000
IL-20	0.961	0.000
Weight (kg)	0.969	0.000
Albumin	0.978	0.000
ST6GAL1	0.980	0.000
CD30	0.988	0.000
ST6GALNAC6	0.992	0.000
G-CSF	0.999	0.000

Supplemental Table 4

Supplemental Table 4. Linear regression results sorted by statistical significance total agalactose (G0) content detected from total plasma IgG N-glycan profiles were correlated to the transcriptomic expression of glycosyltransferases in PBMCs collected on day 0 of hospitalization, clinical data, the relative abundance of anti-nucleocapsid (anti-N) antibodies at visit 1 (day 0 hospitalization) and visit 2 (day 4 of hospitalization), viral RT-qPCR titers, and Luminex data from plasma samples. The coefficient of determination R2 was obtained from linear regression. $p < 0.05$ was considered statistically significant for all tests. Data was sorted by P-value, with $p<0.05$ highlighted in pink.

IgG G0 correlated to:	P value	R^2
ST8SIA6	0.002	0.385
MIF	0.011	0.294
ST3GAL3	0.014	0.264
D-dimer	0.017	0.293
MIP-1b	0.017	0.265
IL-16	0.018	0.262
MCP-1	0.025	0.239
ALG1	0.025	0.227
SCF	0.026	0.246
MANEAL	0.030	0.215
MANEA	0.037	0.199
MAN1A2	0.038	0.199
DPY19L1	0.047	0.183
ALG11	0.048	0.182
AST	0.048	0.190
HGF	0.062	0.180
MCP-3	0.064	0.187
IL-18	0.069	0.163
ALG2	0.073	0.152
ST6GAL1	0.081	0.145
TNF-a	0.085	0.156
MIP-3a	0.086	0.147
DPY19L4	0.096	0.133
Total bilirubin	0.097	0.146
Creatinine	0.108	0.130
Potassium	0.122	0.121
ALG8	0.123	0.115
PIGM	0.126	0.113
BMI (Calc)	0.134	0.109
MAN2A1	0.135	0.108
ST6GALNAC6	0.136	0.108
MAN1B1	0.139	0.106
Weight (kg)	0.149	0.101
MAN2C1	0.151	0.100
MAN1C1	0.153	0.099
IL-13	0.156	0.108
MAN1A1	0.164	0.095
G-CSF	0.171	0.101
ENA-78	0.173	0.106
TMTC4	0.175	0.090
ALG12	0.178	0.089
ST3GAL5	0.195	0.082
POMT1	0.198	0.081
DPM1	0.199	0.081
ALG3	0.219	0.075
TNF-RII	0.231	0.075
BUN	0.233	0.074
MAN2B2	0.234	0.070
IL-12p70	0.239	0.072
MAN2B1	0.240	0.068
IL-15	0.243	0.071
IFNy	0.245	0.070
ALG9	0.246	0.067
ST8SIA1	0.251	0.065
ST6GALNAC3	0.256	0.064
IL-10	0.258	0.071
PIGB	0.263	0.062
DPY19L2	0.268	0.061
PIGZ	0.269	0.061
anti-N IgA Visit 1	0.274	0.063
Sodium	0.276	0.062
ST8SIA3	0.290	0.056
SDF2L1	0.291	0.056
EDEM1	0.298	0.054
ALG1L	0.300	0.053
ST6GALNAC5	0.301	0.053
ddCT_N2_RP Visit 2	0.305	0.062
anti-N IgG Visit 1	0.329	0.050
VEGF-A	0.332	0.050
TMTC3	0.336	0.046
Albumin	0.338	0.051
ST8SIA2	0.348	0.044
Sex	0.351	0.044
PIGV	0.361	0.042
ALG1L2	0.391	0.037
ST6GAL2	0.410	0.034
CD30	0.412	0.036
ST8SIA5	0.412	0.034
Glucose	0.413	0.036
POMT2	0.431	0.031
TMTC1	0.459	0.028
ITAC	0.499	0.024
TSLP	0.499	0.024
C20orf173	0.507	0.022
Serum bicarbonate (HCO3)	0.518	0.022
ST3GAL4	0.534	0.020
IL-6	0.557	0.020
Ferritin	0.561	0.020
ST3GAL1	0.573	0.016
TMTC2	0.611	0.013
Hemoglobin	0.621	0.013
BLC	0.633	0.013
EDEM2	0.647	0.011
ddCT_N1_RP Visit 2	0.658	0.012
ST3GAL2	0.663	0.010
CD40L	0.669	0.011
ST6GALNAC4	0.691	0.008
IL-20	0.699	0.008
ST3GAL6	0.713	0.007
ST6GALNAC1	0.732	0.006
ST6GALNAC2	0.770	0.004
IL-17A	0.778	0.005
CRP	0.782	0.005
DPY19L3	0.783	0.004
MANBA	0.788	0.004
Age	0.801	0.003
ST8SIA4	0.803	0.003
EDEM3	0.819	0.003
MAN2A2	0.820	0.003
Height (cm)	0.826	0.002
anti-N IgM Visit 1	0.918	0.001
ALT	0.994	0.000

Supplemental Table 5

Supplemental Table 5. Linear regression results sorted by statistical significance total di-galactose (G2) content detected from total plasma IgG N-glycan profiles were correlated to the transcriptomic expression of glycosyltransferases in PBMCs collected on day 0 of hospitalization, clinical data, the relative abundance of anti-nucleocapsid (anti-N) antibodies at visit 1 (day 0 hospitalization) and visit 2 (day 4 of hospitalization), viral RT-qPCR titers, and Luminex data from plasma samples. The coefficient of determination R2 was obtained from linear regression. p < 0.05 was considered statistically significant for all tests. Data was sorted by P-value, with p<0.05 highlighted in pink.

IgG G2 correlated to:	P value	R^2
ST8SIA6	0.002	0.383
ST3GAL3	0.009	0.297
MANEAL	0.010	0.285
MAN1A2	0.014	0.268
D-dimer	0.016	0.297
MANEA	0.020	0.243
DPY19L4	0.023	0.233
MIP-3a	0.029	0.228
ALG11	0.029	0.217
DPY19L1	0.030	0.215
IL-16	0.052	0.184
ALG8	0.052	0.175
ALG2	0.056	0.171
BUN	0.061	0.172
ALG1	0.062	0.164
MAN2A1	0.064	0.161
DPM1	0.066	0.159
G-CSF	0.067	0.174
PIGM	0.080	0.145
IL-18	0.081	0.152
Creatinine	0.082	0.151
SCF	0.095	0.147
MIF	0.099	0.137
HGF	0.108	0.137
ST6GAL1	0.110	0.122
MIP-1b	0.111	0.129
PIGB	0.116	0.119
MCP-1	0.125	0.119
MAN1A1	0.136	0.108
MCP-3	0.140	0.124
DPY19L2	0.147	0.102
ST3GAL5	0.147	0.102
ST8SIA1	0.167	0.093
Potassium	0.171	0.096
ALG9	0.179	0.088
ALG1L	0.180	0.088
TMTC4	0.181	0.088
ST6GALNAC5	0.184	0.086
POMT1	0.190	0.084
TMTC3	0.196	0.082
ST8SIA3	0.203	0.080
MAN1C1	0.204	0.079
ddCT_N2_RP Visit 2	0.218	0.088
ALG1L2	0.218	0.075
ITAC	0.227	0.076
ST6GALNAC6	0.238	0.069
VEGF-A	0.240	0.072
ST8SIA2	0.242	0.068
ST6GAL2	0.254	0.064
ST8SIA5	0.255	0.064
MAN1B1	0.264	0.062
BMI (Calc)	0.272	0.060
Albumin	0.274	0.066
ALG3	0.285	0.057
POMT2	0.288	0.056
Hemoglobin	0.299	0.057

IgG G2 correlated to:	P value	R^2
EDEM1	0.307	0.052
ALG12	0.317	0.050
IFNy	0.326	0.051
CD40L	0.329	0.056
TNF-a	0.330	0.053
MAN2C1	0.334	0.047
SDF2L1	0.335	0.047
Weight (kg)	0.336	0.046
C20orf173	0.343	0.045
Total bilirubin	0.343	0.050
ST6GALNAC3	0.357	0.043
PIGV	0.358	0.042
MAN2B2	0.364	0.041
AST	0.387	0.040
TMTC2	0.389	0.037
PIGZ	0.394	0.037
MAN2A2	0.397	0.036
ENA-78	0.408	0.041
Serum bicarbonate (HCO3)	0.429	0.033
Glucose	0.438	0.032
Sodium	0.457	0.029
MAN2B1	0.463	0.027
CD30	0.470	0.028
ST3GAL6	0.471	0.026
BLC	0.487	0.027
CRP	0.488	0.029
anti-N IgA Visit 1	0.505	0.024
ALT	0.513	0.024
IL-12p70	0.516	0.023
anti-N IgG Visit 1	0.524	0.022
ST3GAL2	0.542	0.019
IL-6	0.551	0.020
IL-15	0.562	0.018
IL-10	0.572	0.018
TNF-RII	0.574	0.017
ddCT_N1_RP Visit 2	0.578	0.019
ST8SIA4	0.611	0.013
IL-13	0.646	0.012
EDEM2	0.651	0.010
anti-N IgM Visit 1	0.654	0.011
ST6GALNAC1	0.683	0.009
Age	0.691	0.008
ST3GAL4	0.706	0.007
Ferritin	0.722	0.008
ST6GALNAC4	0.723	0.006
EDEM3	0.730	0.006
ST3GAL1	0.775	0.004
TSLP	0.777	0.004
ST6GALNAC2	0.780	0.004
IL-20	0.801	0.003
TMTC1	0.806	0.003
MANBA	0.861	0.002
DPY19L3	0.898	0.001
Height (cm)	0.918	0.001
IL-17A	0.934	0.000
Sex	0.943	0.000

Supplemental Table 6

Supplemental Table 6. Linear regression results sorted by statistical significance total mono-sialyation (S1) content detected from total plasma IgG N-glycan profiles were correlated to the transcriptomic expression of glycosyltransferases in PBMCs collected on day 0 of hospitalization, clinical data, the relative abundance of anti-nucleocapsid (anti-N) antibodies at visit 1 (day 0 hospitalization) and visit 2 (day 4 of hospitalization), viral RT-qPCR titers, and Luminex data from plasma samples. The coefficient of determination R2 was obtained from linear regression. $p < 0.05$ was considered statistically significant for all tests. Data was sorted by P-value, with $p<0.05$ highlighted in pink.

IgG S1 correlated to:	P value	R^2
ST8SIA6	0.024	0.231
MCP-1	0.026	0.234
MIF	0.027	0.233
MAN1A2	0.029	0.217
IL-16	0.031	0.223
D-dimer	0.036	0.234
MIP-1b	0.038	0.208
MANEAL	0.044	0.188
HGF	0.044	0.206
SCF	0.045	0.206
MANEA	0.047	0.183
MCP-3	0.063	0.189
IL-18	0.079	0.154
DPY19L4	0.082	0.143
ST8SIA1	0.105	0.126
AST	0.117	0.124
ALG11	0.118	0.118
MIP-3a	0.126	0.119
BUN	0.130	0.116
DPY19L2	0.134	0.109
IL-10	0.135	0.120
ST3GAL3	0.142	0.105
ST6GALNAC5	0.145	0.103
ST3GAL4	0.157	0.098
Creatinine	0.158	0.102
ALG9	0.160	0.096
ST8SIA3	0.163	0.095
TNF-a	0.167	0.103
IFNy	0.168	0.098
Potassium	0.175	0.095
ddCT_N2_RP Visit 2	0.175	0.105
VEGF-A	0.188	0.089
ENA-78	0.192	0.098
DPY19L1	0.193	0.083
IL-13	0.195	0.092
IL-6	0.206	0.087
MAN2A1	0.210	0.077
MAN2A2	0.211	0.077
ST8SIA2	0.225	0.073
CD30	0.228	0.076
Serum bicarbonate (HCO3)	0.230	0.075
TMTC3	0.230	0.071
MAN1A1	0.237	0.069
ALG2	0.239	0.069
ALG8	0.246	0.067
Glucose	0.260	0.066
DPM1	0.267	0.061
ST6GAL2	0.268	0.061
ST3GAL5	0.270	0.060
ST6GAL1	0.278	0.059
PIGB	0.278	0.058
ST3GAL2	0.283	0.057
G-CSF	0.284	0.064
TMTC4	0.296	0.054
Sodium	0.296	0.057
ST8SIA5	0.297	0.054

IgG S1 correlated to:	P value	R^2
C20orf173	0.309	0.052
IL-12p70	0.310	0.054
Total bilirubin	0.317	0.056
ALG1	0.319	0.050
TNF-RII	0.327	0.051
MAN1C1	0.339	0.046
CD40L	0.355	0.050
Albumin	0.365	0.046
IL-15	0.374	0.042
TMTC2	0.383	0.038
ddCT_N1_RP Visit 2	0.388	0.044
ST6GALNAC4	0.394	0.036
ST6GALNAC6	0.406	0.035
PIGM	0.414	0.034
MANBA	0.417	0.033
ALG1L2	0.449	0.029
POMT2	0.462	0.027
Sex	0.464	0.027
anti-N IgG Visit 1	0.496	0.025
POMT1	0.497	0.023
TMTC1	0.508	0.022
SDF2L1	0.511	0.022
anti-N IgA Visit 1	0.514	0.023
ALT	0.517	0.024
EDEM1	0.538	0.019
ITAC	0.556	0.019
ALG1L	0.567	0.017
ST6GALNAC3	0.624	0.012
MAN1B1	0.640	0.011
EDEM2	0.654	0.010
anti-N IgM Visit 1	0.663	0.011
DPY19L3	0.690	0.008
BLC	0.706	0.008
ST3GAL6	0.715	0.007
PIGZ	0.722	0.006
Hemoglobin	0.727	0.007
ST8SIA4	0.740	0.006
ALG3	0.741	0.006
ALG12	0.743	0.005
Weight (kg)	0.771	0.004
IL-17A	0.784	0.005
ST6GALNAC2	0.784	0.004
MAN2B2	0.798	0.003
MAN2C1	0.819	0.003
TSLP	0.842	0.002
BMI (Calc)	0.862	0.002
CRP	0.862	0.002
Height (cm)	0.872	0.001
PIGV	0.884	0.001
ST6GALNAC1	0.888	0.001
MAN2B1	0.907	0.001
Age	0.918	0.001
EDEM3	0.922	0.000
Ferritin	0.927	0.001
ST3GAL1	0.959	0.000
IL-20	0.970	0.000

Supplemental Table 7

Supplemental Table 7. COVID cohort trajectories 1-3 (n=12, nonsevere) data was compared to trajectories 4 and 5 (n=10, severe). Transcriptomic expression of glycosyltransferases in PBMCs collected on day 0 of hospitalization, clinical data, relative abundance of anti-nucleocapsid (anti-N) antibodies at visit 1 (day 0 hospitalization) and visit 2 (day 4 of hospitalization), viral RT-qPCR titers, and Luminex data are presented below. Data was analyzed for statistical significance using a two-sided Kruskal-Wallis test. p < 0.05 was considered statistically significant for all tests. Data was sorted by P-value, with p<0.05 highlighted in pink.

	Trajectory 1-3 [median, IQR]	Trajectory 4-5 [median, IQR]	P value
**Total IgM Mann**	26.77 [25.69, 27.46]	19.62 [18.00, 20.80]	0.001
**Total IgM S2**	21.29 [20.53, 23.99]	28.42 [25.55, 31.10]	0.001
**BUN**	17.00 [9.00, 20.50]	43.00 [26.75, 49.50]	0.001
**Trajectory**	2.50 [2.00, 3.00]	5.00 [5.00, 5.00]	0.001
**MAN1A2**	6.04 [5.93, 6.29]	5.56 [5.35, 5.84]	0.007
**Creatinine**	0.79 [0.64, 0.99]	1.54 [1.12, 1.70]	0.011
**D-dimer**	0.81 [0.61, 1.11]	2.77 [1.09, 11.00]	0.011
**Potassium**	3.80 [3.55, 3.90]	4.15 [3.95, 4.50]	0.012
**BLC**	31.01 [13.63, 49.75]	101.17 [51.05, 196.10]	0.014
**Total IgG S1**	15.56 [12.63, 16.91]	12.18 [11.48, 13.68]	0.015
**Total IgG G2**	11.79 [9.93, 13.51]	8.48 [7.82, 9.43]	0.018
**ST3GAL4**	4.33 [4.05, 4.70]	4.73 [4.65, 4.96]	0.018
**ST6GALNAC2**	2.19 [2.06, 2.65]	2.98 [2.62, 3.17]	0.025
**MAN2A1**	5.83 [5.66, 6.11]	5.45 [5.18, 5.67]	0.025
**IL-18**	72.00 [38.20, 163.50]	211.72 [199.12, 274.00]	0.039
**ST6GALNAC5**	1.04 [1.00, 1.31]	1.00 [1.00, 1.00]	0.044
**Total IgG G0**	28.61 [27.14, 37.71]	38.79 [30.93, 42.02]	0.075
**PIGM**	4.64 [4.48, 4.73]	4.41 [4.23, 4.50]	0.075
**ST3GAL5**	5.61 [5.04, 5.98]	5.12 [4.68, 5.44]	0.086
**ALG8**	4.74 [4.50, 5.13]	4.54 [4.37, 4.62]	0.086
**TMTC2**	3.25 [2.76, 3.79]	2.67 [2.42, 3.01]	0.086
**ALG11**	4.09 [3.73, 4.44]	3.70 [3.53, 3.89]	0.086
**Glucose**	112.00 [101.50, 134.00]	142.50 [120.25, 208.25]	0.091
**ALT**	43.50 [23.75, 60.75]	25.00 [11.75, 38.00]	0.096
**POMT2**	3.29 [2.98, 3.62]	2.73 [2.44, 3.22]	0.099
**ALG9**	2.35 [2.13, 2.55]	1.85 [1.60, 2.22]	0.099
**MANEA**	4.16 [3.54, 4.18]	3.56 [3.42, 4.19]	0.099
**MANEAL**	1.98 [1.81, 2.13]	1.72 [1.51, 1.86]	0.099
**CD30**	803.74 [657.12, 1173.84]	1415.00 [902.04, 1437.00]	0.102
**CD40L**	46.52 [29.25, 66.19]	16.35 [11.51, 62.50]	0.111
**SDF2L1**	6.15 [5.76, 6.60]	5.74 [5.52, 6.07]	0.114
**anti-N IgA Visit 1**	3.29 [2.23, 8.63]	11.30 [3.53, 21.97]	0.136
**IL-16**	48.85 [19.96, 81.67]	92.15 [47.24, 303.00]	0.136
**ST8SIA3**	1.03 [1.00, 1.53]	1.00 [1.00, 1.00]	0.169
**AST**	39.00 [30.00, 72.00]	30.00 [25.00, 47.50]	0.18
**Serum bicarbonate (HCO3)**	26.50 [24.35, 29.10]	23.00 [20.55, 27.42]	0.181
**SCF**	8.94 [0.52, 41.50]	8.86 [1.47, 82.00]	0.184
**DPY19L4**	3.28 [2.97, 3.67]	3.07 [2.82, 3.25]	0.187
**Hemoglobin**	13.40 [12.15, 13.75]	12.00 [10.33, 13.55]	0.192
**IFNy**	13.43 [5.98, 32.75]	27.14 [23.00, 34.00]	0.201
**Age**	54.00 [50.00, 72.25]	67.50 [59.00, 75.25]	0.21
**TMTC3**	3.03 [2.62, 3.31]	2.75 [2.48, 2.81]	0.21
**ST8SIA1**	2.07 [1.49, 2.78]	1.56 [1.29, 1.96]	0.235
**ST8SIA4**	6.43 [5.95, 6.60]	6.59 [6.44, 6.94]	0.235
**TMTC4**	4.17 [3.72, 4.54]	3.80 [3.19, 4.36]	0.235
**DPY19L1**	4.43 [4.02, 4.69]	3.98 [3.75, 4.52]	0.235
**CRP**	5.31 [3.34, 11.43]	13.13 [5.33, 14.45]	0.253
**ST6GALNAC1**	1.92 [1.55, 2.95]	2.47 [1.85, 2.95]	0.262
**MCP-1**	54.72 [7.51, 75.10]	115.34 [12.94, 259.00]	0.286
**VEGF-A**	104.25 [57.23, 157.15]	156.50 [135.00, 229.29]	0.286
**ST6GAL1**	7.48 [6.85, 7.82]	6.89 [6.68, 7.27]	0.291
**DPY19L2**	1.48 [1.26, 1.71]	1.24 [1.20, 1.41]	0.291
**ALG2**	5.31 [4.66, 5.48]	5.03 [4.63, 5.26]	0.323
**MAN2A2**	7.49 [7.21, 7.65]	7.67 [7.40, 8.13]	0.323
**MAN1C1**	4.30 [3.71, 5.03]	4.09 [3.19, 4.49]	0.356
**ST6GALNAC4**	5.39 [4.60, 5.87]	4.75 [4.45, 5.40]	0.356
**MANBA**	6.26 [5.74, 6.46]	6.47 [6.01, 6.54]	0.391
**ST8SIA6**	2.34 [2.04, 2.65]	2.15 [1.71, 2.41]	0.391
**MIF**	21.79 [3.09, 91.50]	19.10 [4.38, 239.00]	0.394

	Trajectory 1-3 [median, IQR]	Trajectory 4-5 [median, IQR]	P value
**ST8SIA5**	1.26 [1.05, 2.08]	1.11 [1.03, 1.48]	0.427
**DPM1**	4.97 [4.81, 5.10]	4.79 [4.49, 5.08]	0.429
**MAN1A1**	7.03 [6.63, 7.61]	6.98 [6.55, 7.05]	0.429
**POMT1**	5.07 [4.69, 5.15]	4.56 [4.16, 5.11]	0.429
**anti-N IgM Visit 1**	4.52 [3.61, 7.11]	6.19 [4.79, 8.95]	0.44
**ST8SIA2**	1.00 [1.00, 1.62]	1.00 [1.00, 1.03]	0.467
**ALG1**	4.73 [4.55, 4.93]	4.58 [4.24, 4.80]	0.468
**ST6GALNAC3**	3.05 [2.83, 3.80]	3.65 [2.93, 4.10]	0.468
**IL-6**	13.73 [10.21, 19.50]	15.50 [1.56, 72.00]	0.47
**HGF**	51.00 [18.01, 89.45]	127.50 [14.20, 254.00]	0.47
**ST6GAL2**	1.02 [1.00, 1.82]	1.00 [1.00, 1.06]	0.471
**TNF-RII**	64.50 [40.32, 158.19]	82.41 [54.54, 192.00]	0.477
**Sodium**	139.00 [135.50, 141.50]	138.50 [133.00, 141.75]	0.478
**ddCT_N1_RP Visit 2**	2.58 [1.16, 5.69]	4.72 [2.85, 6.42]	0.483
**C20orf173**	1.06 [1.00, 1.21]	1.13 [1.04, 1.14]	0.505
**ST3GAL3**	3.96 [3.31, 4.16]	3.66 [3.23, 3.96]	0.51
**EDEM1**	6.47 [6.17, 7.06]	6.30 [6.11, 6.79]	0.51
**Albumin**	3.30 [3.23, 3.55]	3.10 [2.73, 3.58]	0.519
**MCP-3**	18.74 [10.83, 28.03]	21.00 [5.46, 53.76]	0.54
**Height (cm)**	165.05 [159.38, 177.80]	167.55 [163.52, 177.18]	0.551
**TMTC1**	3.88 [3.14, 4.49]	3.90 [2.42, 4.32]	0.553
**ALG3**	5.94 [5.75, 6.14]	5.97 [5.51, 6.02]	0.553
**ALG1L2**	1.55 [1.37, 1.80]	1.43 [1.32, 1.58]	0.553
**IL-13**	4.61 [1.56, 15.00]	15.36 [1.32, 16.25]	0.563
**TNF-a**	11.00 [7.14, 17.00]	13.50 [11.52, 14.81]	0.569
**anti-N IgG Visit 1**	3.54 [1.84, 7.39]	4.60 [1.57, 35.64]	0.57
**BMI (Calc)**	35.97 [31.33, 38.79]	32.88 [24.93, 40.27]	0.598
**ST3GAL6**	4.55 [3.98, 4.84]	4.42 [4.19, 4.50]	0.598
**PIGB**	4.31 [4.18, 4.48]	4.22 [3.89, 4.53]	0.598
**Ferritin**	742.50 [306.75, 1609.50]	693.00 [581.00, 794.00]	0.624
**ddCT_N2_RP Visit 2**	1.56 [0.16, 4.68]	4.17 [0.12, 5.66]	0.642
**MIP-3a**	19.50 [13.75, 92.27]	46.00 [15.50, 95.72]	0.644
**ST3GAL2**	6.20 [5.91, 6.68]	6.55 [6.06, 6.64]	0.644
**DPY19L3**	4.09 [3.91, 4.49]	4.28 [3.75, 4.62]	0.644
**ALG12**	4.55 [4.21, 4.62]	4.29 [4.11, 4.63]	0.644
**MIP-1b**	8.77 [1.70, 40.12]	5.81 [3.04, 79.00]	0.67
**IL-20**	21.88 [9.30, 34.50]	9.48 [4.91, 35.00]	0.67
**IL-10**	8.75 [1.29, 17.37]	11.81 [7.57, 16.75]	0.671
**ST3GAL1**	7.03 [6.42, 7.32]	7.02 [6.53, 7.29]	0.692
**TSLP**	12.05 [6.10, 18.88]	13.50 [3.52, 19.00]	0.722
**MAN2C1**	6.29 [5.99, 6.57]	5.91 [5.69, 6.77]	0.742
**Weight (kg)**	97.20 [90.32, 111.00]	88.55 [76.30, 112.13]	0.792
**ENA-78**	135.69 [58.70, 217.50]	148.95 [96.22, 225.56]	0.804
**G-CSF**	16.00 [9.59, 19.50]	15.00 [11.00, 32.12]	0.82
**IL-12p70**	2.37 [2.07, 15.12]	2.73 [1.74, 12.00]	0.831
**ITAC**	39.22 [21.72, 114.88]	76.00 [24.00, 95.50]	0.831
**IL-15**	4.14 [2.68, 20.00]	4.06 [2.40, 15.00]	0.831
**PIGV**	4.41 [3.94, 4.86]	4.48 [4.21, 4.57]	0.843
**PIGZ**	1.93 [1.81, 2.40]	1.96 [1.87, 2.20]	0.843
**ST6GALNAC6**	5.67 [4.91, 6.33]	5.30 [4.84, 5.95]	0.843
**MAN1B1**	6.13 [5.55, 6.29]	5.79 [5.51, 6.36]	0.843
**Total bilirubin**	0.55 [0.40, 0.60]	0.50 [0.40, 0.78]	0.848
**IL-17A**	19.79 [16.57, 31.98]	18.40 [15.75, 32.69]	0.869
**EDEM2**	6.59 [6.38, 6.91]	6.70 [6.34, 6.79]	0.895
**EDEM3**	6.18 [6.07, 6.30]	6.19 [5.77, 6.59]	0.947
**ALG1L**	1.11 [1.06, 1.22]	1.15 [1.03, 1.29]	0.974
**MAN2B2**	6.40 [6.04, 6.74]	6.41 [5.92, 6.72]	1
**MAN2B1**	7.28 [6.67, 7.78]	7.27 [6.80, 7.70]	1

Supplemental Table 8

Supplemental Table 8. Spike S1-specific IgG N-glycans profile peaks from pooled COVID cohort trajectories 1-3 (nonsevere) was compared to trajectories 4 and 5 (severe). Area under the curve analysis revealed differences in N-glycan peak abundance. Fold difference is calculated by dividing the severe N-glycan peak abundance by the nonsevere N-glycan peak abundance respectively. GP = Glycan Peak.

		% Area		Fold
GP#	Spike S1-Specific IgG	Severe	Nonsevere	Difference
1	FA1G0	0.774	0.158	4.9
2	A2G0	1.382	1.668	0.8
3	FA2G0	35.245	17.705	2.0
4	FA2BG0	2.499	4.638	0.5
5	A2G1 (1,6)	1.205	2.266	0.5
6	A2G1 (1,3)	0.359	0.674	0.5
7	FA2G1 (1,6)	22.231	13.363	1.7
8	FA2G1 (1,3)	7.828	5.891	1.3
9	FA2BG1 (1,6)	1.553	3.196	0.5
10	FA2BG1 (1,3)	0.588	0.493	1.2
11	A2G2	0.550	1.856	0.3
12	FA2G2	6.925	9.487	0.7
13	FA2BG2	1.183	1.179	1.0
14	FA2G1S1 (1,6)	0.205	0.278	0.7
15	FA2G1S1 (1,3)	1.967	2.409	0.8
16	A2G2S1 (1,6)	0.368	0.395	0.9
17	A2G2S1 (1,3)	1.277	3.536	0.4
18	FA2G2S1	4.562	8.436	0.5
19	FA2BG2S1	0.581	0.905	0.6
20	Unidentified	0.217	0.913	0.2
21	A2G2S2 (1,3)	7.095	17.322	0.4
22	A2BG2S2	0.396	0.320	1.2
23	FA2G2S2	0.724	2.250	0.3
24	FA2BG2S2	0.287	0.662	0.4

Supplemental Table 9

Supplemental Table 9. Spike S1-specific IgM N-glycans profiles from pooled COVID cohort trajectories 1-3 (nonsevere) was compared to trajectories 4 and 5 (severe). Area under the curve analysis revealed differences in N-glycan peak abundance. Fold difference is calculated by dividing the severe N-glycan peak abundance by the nonsevere N-glycan peak abundance respectively. GP = Glycan Peak.

		% Area		Fold
GP #	Spike S1-specific IgM	Severe	Nonsevere	Difference
1	A1	0.142	0.235	0.6
2	FA1	0.184	0.177	1.0
3	M4G1	0.422	0.389	1.1
4	FA2	0.667	0.519	1.3
5	FM4A1	0.466	0.372	1.3
6	M5	5.381	6.078	0.9
7	FA2G1	0.415	0.201	2.1
8	M5A1	0.907	1.417	0.6
9	FA2BG1	1.865	1.157	1.6
10	M6 D1/D2	3.362	3.320	1.0
11	M6 D3	5.846	7.138	0.8
12	A2G2	0.092	0.179	0.5
13	FA2G2	5.333	3.356	1.6
14	FA2BG2	1.653	1.269	1.3
15	FA2G1S1/M7	3.204	2.989	1.1
16	FA2BG1S1	1.968	1.426	1.4
17	A2G2S1	2.985	2.894	1.0
18	FA2G2S1	18.256	13.282	1.4
19	M8	2.600	2.826	0.9
20	FA2BG2S1	8.327	11.417	0.7
21	A2G2S2 *(α-2,3)*	1.194	1.985	0.6
22	FA2G2S2 *(α-2,3)*	0.508	0.578	0.9
23	A2G2S2	21.720	25.036	0.9
24	FA2G2S2	6.380	5.250	1.2
25	FA2BG2S2	1.922	3.537	0.5
26	M10	1.225	0.626	2.0
27	A3G3S2/FA3G3S2	0.191	0.119	1.6
28	A3G3S2/FA3G3S2	0.285	0.284	1.0
29	FA3G3S2	0.065	0.077	0.8
30	A3G3S3 (6,6,6)	0.562	0.551	1.0
31	A3G3S3 (6,6,3)	0.303	0.254	1.2
32	A3G3S3 (6,3,3)	0.532	0.729	0.7
33	A3G3S3 (3,3,3)	1.039	0.337	3.1
30-33	Summation of S3	2.435	1.871	1.3

Supplemental Table 10

Supplemental Table 10. Sialidase-digested Spike S1-specific IgM N-glycans profiles from pooled COVID cohort trajectories 1-3 (nonsevere) was compared to trajectories 4 and 5 (severe). Area under the curve analysis revealed differences in N-glycan peak abundance. Fold difference is calculated by dividing the severe N-glycan peak abundance by the nonsevere N-glycan peak abundance respectively. GP = Glycan Peak.

	Sialidase-digested	% Area		Fold
GP#	Spike S1-specific IgM	Severe	Nonsev	Difference
1	**M4G1**	0.243	0.228	1.1
2	**FA2G0**	0.281	0.429	0.7
3	**FM4A1**	0.888	0.538	1.7
4	**M5**	4.953	6.282	0.8
5	**A2G1**	3.118	1.644	1.9
6	**M5A1**	2.225	2.327	1.0
7	**FA2BG1**	1.762	1.694	1.0
8	**M6 D1/D2**	4.656	4.694	1.0
9	**M6 D3**	4.413	5.335	0.8
10	**A2G2**	21.102	27.590	0.8
11	**FA2G2**	20.346	16.812	1.2
12	**FA2BG2**	10.229	15.449	0.7
13	**M7**	1.279	1.521	0.8
14	**M7**	3.980	2.872	1.4
15	**A3G3**	5.177	2.706	1.9
16	**M8**	1.127	1.404	0.8
17	**M8**	4.879	1.982	2.5
18	**M8**	4.333	3.125	1.4
19	**M8**	0.932	0.741	1.3
20	**M9**	0.611	0.480	1.3
21	**M9**	1.920	1.399	1.4
22	**M10**	1.544	0.749	2.1
