# Supplementary material for: IgM N-glycosylation correlates with COVID-19 severity and rate of complement deposition
Source: Nat Commun. 2024 Jan 9;15:404. doi: 10.1038/s41467-023-44211-0 (PMC10776791; doi:10.1038/s41467-023-44211-0)
Supplement: Supplementary file 3 — Reporting Summary [file 41467_2023_44211_MOESM3_ESM.pdf]

Corresponding author(s): Mary Ann Comunale, Elias Haddad

Last updated by author(s): Nov 7, 2023

Reporting Summary

Nature Portfolio wishes to improve the reproducibility of the work that we publish. This form provides structure for consistency and transparency in reporting. For further information on Nature Portfolio policies, see our Editorial Policies and the Editorial Policy Checklist.

Statistics

For all statistical analyses, confirm that the following items are present in the figure legend, table legend, main text, or Methods section.

n/a	Confirmed	
☐	☒	The exact sample size (*n*) for each experimental group/condition, given as a discrete number and unit of measurement
☐	☒	A statement on whether measurements were taken from distinct samples or whether the same sample was measured repeatedly
☐	☒	The statistical test(s) used AND whether they are one- or two-sided<br>*Only common tests should be described solely by name; describe more complex techniques in the Methods section.*
☒	☐	A description of all covariates tested
☐	☒	A description of any assumptions or corrections, such as tests of normality and adjustment for multiple comparisons
☐	☒	A full description of the statistical parameters including central tendency (e.g. means) or other basic estimates (e.g. regression coefficient) AND variation (e.g. standard deviation) or associated estimates of uncertainty (e.g. confidence intervals)
☐	☒	For null hypothesis testing, the test statistic (e.g. *F*, *t*, *r*) with confidence intervals, effect sizes, degrees of freedom and *P* value noted<br>*Give P values as exact values whenever suitable.*
☒	☐	For Bayesian analysis, information on the choice of priors and Markov chain Monte Carlo settings
☒	☐	For hierarchical and complex designs, identification of the appropriate level for tests and full reporting of outcomes
☒	☐	Estimates of effect sizes (e.g. Cohen's *d*, Pearson's *r*), indicating how they were calculated

Our web collection on statistics for biologists contains articles on many of the points above.

Software and code

Policy information about availability of computer code

Data collection	Microsoft Excel v16.66.1, Waters Empower v3, R Studio v4.2.3, FacsDiva v9.0, and FlowJo v10.9 software were used to collect data
Data analysis	Microsoft Excel v16.66.1, GraphPad Prism v8, Waters Empower v3, R Studio v4.2.3, and FlowJo v10.9 software were used to analyze data

For manuscripts utilizing custom algorithms or software that are central to the research but not yet described in published literature, software must be made available to editors and reviewers. We strongly encourage code deposition in a community repository (e.g. GitHub). See the Nature Portfolio guidelines for submitting code & software for further information.

Data

Policy information about availability of data

All manuscripts must include a data availability statement. This statement should provide the following information, where applicable:

- Accession codes, unique identifiers, or web links for publicly available datasets
- A description of any restrictions on data availability
- For clinical datasets or third party data, please ensure that the statement adheres to our policy

All IMPACC data including those generated in this study have been deposited in the Immunology Database and Analysis Portal (ImmPort), a NIAID Division of Allergy, Immunology and Transplantation funded data repository under accession code SDY1760 (immport.org). All raw and processed data are available under restricted access to comply with the NIH public data sharing policy for IRB-exempted public health surveillance studies, Access can be obtained via AccessClinicalData@NIAID

(https://accessclinicaldata.niaid.nih.gov/study-viewer/clinical_trials). Additional guidelines for access are outlined on ImmPort (https://docs.immport.org/home/impaccslides). The source data generated in the figures are provided as a Source Data file.

Research involving human participants, their data, or biological material

Policy information about studies with human participants or human data. See also policy information about sex, gender (identity/presentation), and sexual orientation and race, ethnicity and racism.

Reporting on sex and gender	This study collected information on participant sex as noted within the electronic medical record.
Reporting on race, ethnicity, or other socially relevant groupings	This study collected information on participant race and ethnicity as noted within the electronic medical record. For more information, please see the manuscript published in Science Immunology in 2021 "Immunophenotyping assessment in a COVID-19 cohort (IMPACC): A prospective longitudinal study" detailing the data collection approach: https://www.science.org/doi/10.1126/sciimmunol.abf3733
Population characteristics	This study collected biological samples from patients with COVID-19, and stratifies the cohort into five trajectories as determined in: "Immunophenotyping assessment in a COVID-19 cohort (IMPACC): A prospective longitudinal study" (https://www.science.org/doi/10.1126/). Cohort demographics of the COVID-19 patients, including age, sex, race, ethnicity, comorbitiy, body mass index, time from symptom onset to hospitalization for COVID-19 infection, levels of respiratory support, SOFA score, prescription of Remdesivir, and clinical laboratory assessment of D-dimer, BUN, creatinine, and potassium as noted within the electronic medical record are presented in Table 1. On average, the patient population was 61.6 years old, 60% male, 86% white, 68% non-hispanic, with an majority of patients having a body-mass-index (BMI) in the 30-39.9 range.
Recruitment	This study recruited participants admitted to the hospital and diagnosed with an acute case of COVID-19 within the first 48 hours of admission. Participants enrolled under the public health surveillance exclusion were provided information sheets describing the study, samples to be collected, and plans for data de-identification, and use. Those that requested not to participate after reviewing the information sheet were not enrolled. In addition, participants did not receive compensation for study participation while inpatient, and subsequently were offered compensation during outpatient follow-ups. For more information, please see the "Immunophenotyping assessment in a COVID-19 cohort (IMPACC): A prospective longitudinal study" detailing the sample collection approach (https://www.science.org/doi/10.1126/sciimmunol.abf3733)
Ethics oversight	This study was approved by Drexel's IRB Protocols 2004007753 and 2102008337.

Note that full information on the approval of the study protocol must also be provided in the manuscript.

Field-specific reporting

Please select the one below that is the best fit for your research. If you are not sure, read the appropriate sections before making your selection.

☒ Life sciences ☐ Behavioural & social sciences ☐ Ecological, evolutionary & environmental sciences

For a reference copy of the document with all sections, see nature.com/documents/nr-reporting-summary-flat.pdf

Life sciences study design

All studies must disclose on these points even when the disclosure is negative.

Sample size	The patient cohort was limited by the n=10 severe COVID-19 (trajectory 4+5) Day 4 patient plasma available. We then compiled a nonsevere COVID-19 Day 4 (n=12) cohort with similar distribution of age, sex, and race to that of the severe cohort. In addition, Day 7 severe (n=6) and nonsevere (n=10) COVID-19 cohorts consisted of the remaining available plasma from patient cohorts who had continued to participate in the study, excluding those who had deceased from COVID-19 prior to the Day 7 collection time point.
Data exclusions	The only cause for a clinical parameter or assayed biomarker to be excluded was due to dataset incompleteness. Any datasets missing greater than 3 of 22 values were excluded from analysis to reduce potential bias due to incomplete datasets as advised in Dong et al. 2013.
Replication	Analysis of immunoglobulin N-glycan profiles and complement deposition assays were experimentally repeated, and replication was successful. Clinical data, transcription data, and other parameters were collected in accordance with industry standards and these data have previously been approved for publication through prior IMPACC-sponsored manuscripts.
Randomization	Patient samples were allocated into experimental groups using the IMPACC study trajectory stratification. The goal of this work was to determine if the N-glycans associated with immunoglobulin G and M differed across these groups. During specific experiments, the order of samples analyzed via UPLC-FLR-ESI-MS was varied to avoid any column-specific effects across the cohorts.
Blinding	Blinding of the samples during analysis was not applicable to this study as the randomized experimental methods used to analyze complement deposition and N-glycan profiles did not provide opportunity for experimental-bias or participant bias.

Reporting for specific materials, systems and methods

We require information from authors about some types of materials, experimental systems and methods used in many studies. Here, indicate whether each material, system or method listed is relevant to your study. If you are not sure if a list item applies to your research, read the appropriate section before selecting a response.

Materials & experimental systems

n/a	Involved in the study
☐	☒ Antibodies
☒	☐ Eukaryotic cell lines
☒	☐ Palaeontology and archaeology
☒	☐ Animals and other organisms
☒	☐ Clinical data
☒	☐ Dual use research of concern
☒	☐ Plants

Methods

n/a	Involved in the study
☒	☐ ChIP-seq
☐	☒ Flow cytometry
☒	☐ MRI-based neuroimaging

Antibodies

Antibodies used

IgM Isolation: Anti-Human IgM (μ-chain specific)–Agarose antibody produced in goat (Sigma, A9935-5ML, Lot#: 0000188278)
Flow Cytometry: 1:100 FITC labeled Goat anti-Guinea pig Complement C3 antibody (MP Biomedicals, 55385, Lot #:08077).
Western blot for method development of IgG isolation: 1:10,000 Goat anti-human IgG IR680LT (LiCor, 926-68032, Lot #:D00421-13).
Western blot for method development of IgM isolation: 1:5,000 Anti-Human IgM (μ-chain specific) antibody produced in goat (Sigma, I2386-1ML, Lot #: 118M4782V), 1:5,000 Donkey anti-Goat IgG IR800CW (LiCor, 926-32214, Lot# B70416-02).

Validation

Anti-Human IgM (μ-chain specific)–Agarose antibody produced in goat (Sigma, A9935-5ML, Lot#: 0000188278). Validation Statement: https://www.sigmaaldrich.com/US/en/product/sigma/a9935

The FITC labeled Goat anti-Guinea pig Complement C3 antibody antibody was validated internally during the complement deposition assays by performing Flow-minus-one (FMO) to confirm the antibody does not bind non-specifically to elements other than deposited Guinea pig C3. Furthermore, this antibody has been employed in complement deposition assays developed in the Galit Alter lab in 2019. DOI: 10.1016/j.jim.2019.07.002. Validation Statement: https://www.mpbio.com/media/document/file/coa/dest/5/5/3/8/5/0/55385-08077.pdf

Goat anti-human IgG IR680LT (LiCor, 926-68032, Lot #: D00421-13) Validation Statement: https://www.licor.com/documents/x0uq5og5eokv4jzrmmkidk3b76yuim6w

Anti-Human IgM (μ-chain specific) antibody produced in goat (Sigma, I2386-1ML, Lot #: 118M4782V) https://www.sigmaaldrich.com/certificates/Graphics/COfAInfo/sigmail01/pdf/I2386_SPEC.pdf

Donkey anti-Goat IgG IR800CW (LiCor, 926-32214, Lot# B70416-02) Validation Statement: https://www.licor.com/documents/8w2snwg1ztrbpstg9umhh0cyr7unm1l1

Flow Cytometry

Plots

Confirm that:

☒ The axis labels state the marker and fluorochrome used (e.g. CD4-FITC).

☒ The axis scales are clearly visible. Include numbers along axes only for bottom left plot of group (a 'group' is an analysis of identical markers).

☒ All plots are contour plots with outliers or pseudocolor plots.

☒ A numerical value for number of cells or percentage (with statistics) is provided.

Methodology

Sample preparation

We analyzed the deposition of complement onto a FluoSpheres™ NeutrAvidin™-Labeled Microspheres presenting the RBD or Spike S1 antigen to patient antibodies, adapted from the complement deposition assay protocol developed by the Galit Alter lab in 2019. DOI: 10.1016/j.jim.2019.07.002

Instrument

Fortessa Flow Cytometer (BD)

Software

We used FacsDiva v9.0 and FlowJo v10.9 for flow cytometry analysis

Cell population abundance

We did not analyze cells, rather we analyzed the abdunance of complement deposition on FluoSpheres™ NeutrAvidin™-Labeled Microspheres as adapted from the complement deposition assay protocol developed by the Galit Alter lab in 2019. DOI: 10.1016/j.jim.2019.07.002.

Gating strategy	We followed the gating strategy adapted from the complement deposition assay protocol developed by the Galit Alter lab in 2019. DOI: 10.1016/j.jim.2019.07.002. Please see figure 6a for an example of the gating strategy.

☐ Tick this box to confirm that a figure exemplifying the gating strategy is provided in the Supplementary Information.
